# Supplementary material for: Causal interplay between lactose intolerance and gut microbiota: a combined bidirectional Mendelian randomization and in vivo validation study
Source: Front Nutr. 2026 Jun 1;13:1803337. doi: 10.3389/fnut.2026.1803337 (PMC13265576; doi:10.3389/fnut.2026.1803337)
Supplement: Supplementary file 1 [file Data_Sheet_1.zip › supplementary materials/Forward/leave one out cross validation/ebi-a-GCST90027813.finngen_R12_E4_LACTONAS.pdf]

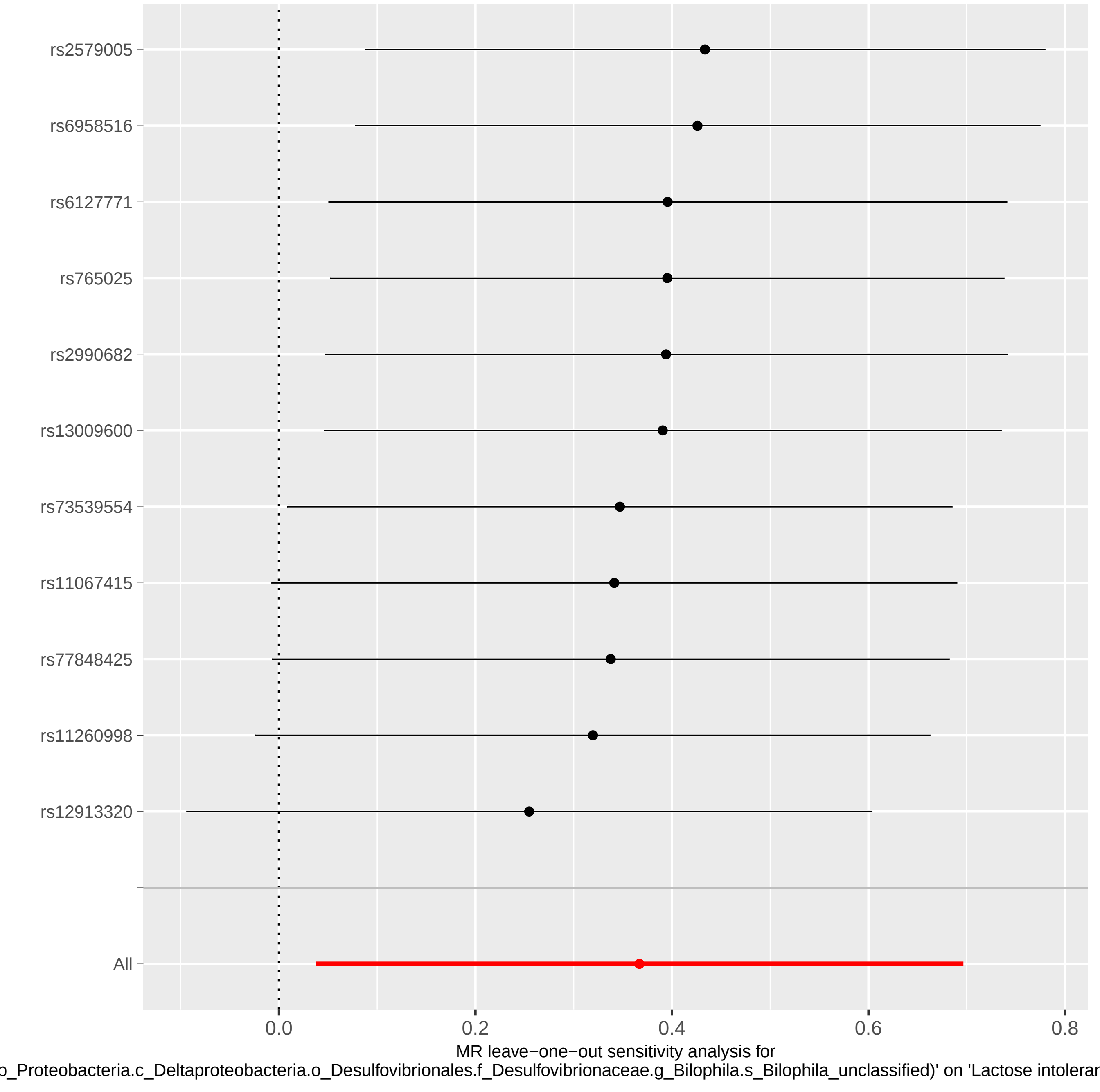

rs2579005
rs6958516
rs6127771
rs765025
rs2990682
rs13009600
rs73539554
rs11067415
rs77848425
rs11260998
rs12913320
All
0.0
0.2
0.4
0.6
0.8
MR leave−one−out sensitivity analysis for
p_Proteobacteria.c_Deltaproteobacteria.o_Desulfovibrionales.f_Desulfovibrionaceae.g_Bilophila.s_Bilophila_unclassified)' on 'Lactose intoleran
